# Safety and Efficacy of Eucaloric Very Low-Carb Diet (EVLCD) in Type 1 Diabetes: A One-Year Real-Life Retrospective Experience

**DOI:** 10.3390/nu14153208

**Published:** 2022-08-05

**Authors:** Andrea Kleiner, Barbara Cum, Livia Pisciotta, Ivan Raffaele Cincione, Ludovica Cogorno, Amalia Prigione, Antonio Tramacere, Andrea Vignati, Luca Carmisciano, Samir Giuseppe Sukkar

**Affiliations:** 1 Salus AlpeAdria Diabetes Center, 33100 Udine, Italy; 2 Dietetics and Clinical Nutrition Unit, IRCCS Policlinic Hospital San Martino, 16132 Genoa, Italy; 3 Department of Internal Medicine, University of Genoa, 16132 Genoa, Italy; 4 Department of Clinical and Experimental Medicine, University of Foggia, 71122 Foggia, Italy; 5 Department of Experimental Medicine-Medical Pathophysiology, Food Science and Endocrinology Section, Sapienza University of Rome, 00185 Rome, Italy; 6 Department of Health Sciences (DISSAL), Section of Biostatistics, University of Genoa, Via Pastore, 16132 Genoa, Italy

**Keywords:** type 1 diabetes, DM1, eucaloric very low-carb diet, EVLCD, safety

## Abstract

A eucaloric very low carbohydrate diet (EVLCD) is a diet with a daily caloric intake equal to the total daily energy expenditure (TDEE) with a carbohydrate content of <50 g/day. The literature on very low carbohydrate diets (VLCD) in type 1 diabetes (DM 1) is limited, although recently published scientific studies have highlighted their safety and efficacy in managing DM 1. In this retrospective analysis, we report the clinical data of 33 patients affected by DM 1 carrying out insulin therapy who switched voluntarily from their usual diet (high carb, low fat) to an EVLCD. Our aim is to evaluate the glycemic control, the amount of insulin needed in order to maintain glycemic control and safety of EVLCD. The switch improved glycemic control (mean glycated hemoglobin decreased from 8.3% to 6.8% (*p* < 0.01). The number of patients who reached a glycated hemoglobin value of <7% increased statistically from 12% to 57% (*p* < 0.01), and there was a statistically significant decrease (*p* < 0.01) in the units of daily insulin (from 36.7± 14.9 IU to 28.9 ±9.1 IU) A reduction from 54% to 24% in clinical level 2 hypoglycemia episodes was reported. No cases of severe hypoglycemia or ketoacidosis were observed. The results of the study support that EVLCD in DM 1 seems safe and effective when adopted under tight medical supervision.

## 1. Introduction

Type 1 diabetes (DM 1) is a chronic disease characterized by the immune mediated related destruction of pancreatic beta-cells; the destruction of the beta-cells leads to a substantially absolute insulin deficiency and to a scenario in which the residual pancreatic microsecretory activity is insufficient for glucose homeostasis [1,2]. The disease causes hyperglycemia secondary to insulin deficiency, which becomes lethal over time without insulin replacement therapy [1,2]. Before 1922, in the absence of available pharmacological treatments, DM 1 was treated with a low-carb diet only, which allowed survival for just a few years without insulin [3]; since 1922, the disease has been treated with exogenous insulin injections, which has drastically increased the survival of patients and decreased the incidence of complications. From a pharmacological point of view, because the hallmark of DM 1 is the absence or near absence of beta-cell function, the use of insulin replacement therapy, through multiple subcutaneous injections of insulin, is necessary [4]. From a nutritional perspective, although evidence suggests that there is not an ideal percentage of calorie from carbohydrate, fat and protein for people with DM 1, DM 1 is often clinically managed with a Mediterranean diet in association with carbohydrate counting [5]. Despite the extraordinary pharmacological and technological progress that has occurred in almost a century of diabetes treatment, the achievement of glycemic targets is still difficult for most of these patients [6,7]. To date, a minority of patients (approximately 28% in Italy) reaches the suggested target of glycated hemoglobin value below 7.0% [8].

In terms of complications, DM 1 remains one of the main risk factors for the development of microangiopathies [9]—such as retinopathy, nephropathy, and neuropathy—and represents a major cardiovascular risk factor for the development of vasculopathy, coronary heart disease, stroke and early cardiovascular death [9,10]. Furthermore, the issue of acute DM 1 complications—such as hypoglycemia [11,12] and diabetic ketoacidosis [13] remains unsolved. These complications represent one of the leading causes of hospitalization [14,15] with high related social costs [16], and unfortunately, even today, they remain one of the possible causes of death for young and older adults with diabetes, for both type 1 and type 2 [17,18].

Although there is still no definitive consensus on their definition, low-carbohydrate diets are generally defined as diets with a carbohydrate content of less than 130 g per day [19]. Very low-carbohydrate diets (VLCD) represent a subgroup of low-carb diets characterized by high fat, moderate protein, and very low carbohydrate content (<50 gr).

Cases of patients treated with insulin and low carbohydrate diets (LCD) have been reported since the 1970s [20] but traditionally, these diets have been contraindicated for patients with DM 1 because of the risk of the onset of diabetic ketoacidosis or hypoglycemia related to the reduced carbohydrate intake [21,22]. Moreover, the long-term outcomes of very low carbohydrate diets in people with DM 1 are unknown, and there is still debate about the safety and tolerability [23].

Recently, however, several scientific studies have been published highlighting the safety and efficacy of low carb diets in the management of DM 1, showing better glycemic control and reductions in insulin requirement, hypoglycemia rates, and reductions in the incidence of diabetic ketoacidosis [21,22,23,24].

Our aim is to evaluate glycemic control, the amount of insulin needed to maintain glycemic control and safety of an eucaloric very low-carb diet (EVLCD) in patients suffering from DM 1 who previously had a standard diet in a real-life contest. EVLCD is characterized by a carbohydrate content <50 g/die and by a caloric intake equal to the subject’s energy needs.

## 2. Materials and Methods

This is a retrospective, real-life study on patients suffering from DM 1 belonging to hospitals of Azienda Sanitaria Locale of Udine (Friuli Venezia Giulia, Italy) and taken in charge at the clinic of Dr. Kleiner.

The case series concerns 33 patients with DM 1 regularly treated with insulin therapy, who decided, voluntarily, to switch from their usual diet rich in carb (high carb, low fat) to an EVLCD for a period of 12 months from December 2017.

Data collected during the period of observation were personal and anthropometric data, the physical examination, the food anamnesis, and laboratory tests (blood count, HbA1c%, total cholesterol, HDL, triglycerides, creatinine, aspartate transaminase, alanine transaminase, and insulinemia).

Personal data, anthropometric data, the physical examination, and laboratory tests were collected at baseline and at the endline during outpatient practice using Excel.

Self-measurement of blood glucose and of ketonemia were recorded by patients at home.

The retrospective analysis of the data was approved by the individuals themselves through written consent and authorization.

According to the medical recording, the following parameters were also evaluated: cardiovascular (CV) risk factors (history of major cardiovascular events, smoking, hypertension and atherosclerotic diseases), CV events (myocardial infarction and stroke) and microvascular complications (retinopathy, nephropathy, neuropathy).

Level 2 hypoglycemia was defined as blood sugar levels <54 mg/dL.

**Figure 1:**
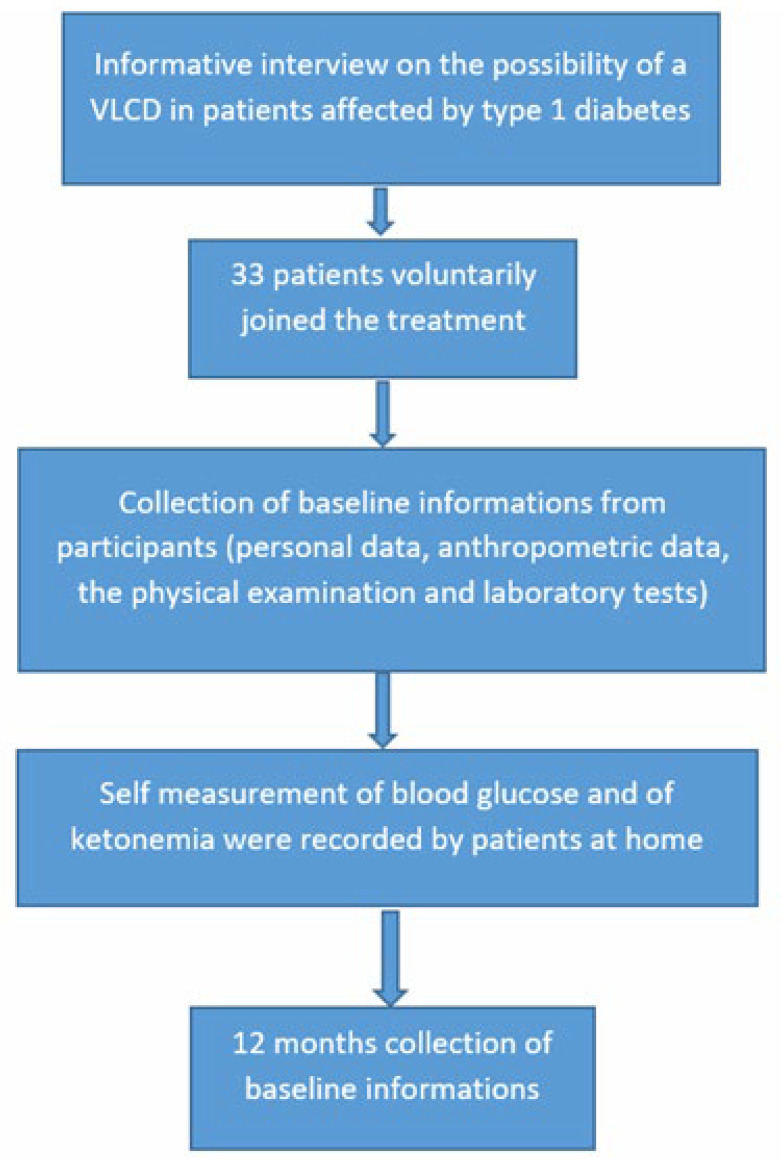
Flow chart of the study.

Severe hypoglycemia was defined as a hypoglycemic value requiring assistance for the treatment. A flow chart of the study is shown in Figure 1.

### 2.1. Anthropometric Measures

Bodyweight was measured to the nearest 0.1 kg using an electronic scale (Seca srl), and height to 1 cm using a wall-mounted portable stadiometer (Seca srl). Body mass index (BMI) was calculated in kg/m^2^.

### 2.2. Blood Exams

All laboratory tests were performed by a certified laboratory belonging to hospitals of Azienda Sanitaria Locale (Friuli Venezia Giulia, Italy). The HbA1c was determined with immunoassay, using fasting venous samples collected by a hospital team. It was performed twice a year if glycemic values were at target, or every three months if not at target.

Total cholesterol and HDL were determined with the enzymatic colorimetric assay in homogenous phase (Roche Cobas e 702, Roche Diagnostics, Mannheim, Germany), triglycerides by an enzymatic colorimetric method (Roche Cobas e 702, Roche Diagnostics, Mannheim, Germany). The glomerular filtration rate was estimated through the CKD-EPI formula, and low-density lipoprotein (LDL) cholesterol was calculated using the Friedewald formula.

Moreover, strict blood glucose home monitoring was performed by patients through glucometers (Glucomen LX- Menarini Diagnostics) on fresh capillary whole blood by puncture with lancet, together with the use of a continuous glucose monitoring (CGM) sensors (Freestyle Libre Abbott Laboratories, North Chicago, IL, USA).

The glucose measurement was performed before and after every meal (breakfast, lunch and dinner). The dosage of blood beta-hydroxybutyrate with Glucomen ketostrips was suggested in order to exclude pathological values. Glycemia values were weekly communicated to the physicians (by telephone or messaging applications).

### 2.3. Eating Pattern

The subjects included in this case series decided to switch from their usual diet high in carbohydrates (>200 gr/die) on average composed of 55% carbohydrates, 25% proteins and 20% fats to a EVLCD composed of an average of 70% fats, 25% proteins and 5% carbohydrates (carbohydrates intake <50 g/day).

**Figure 2:**
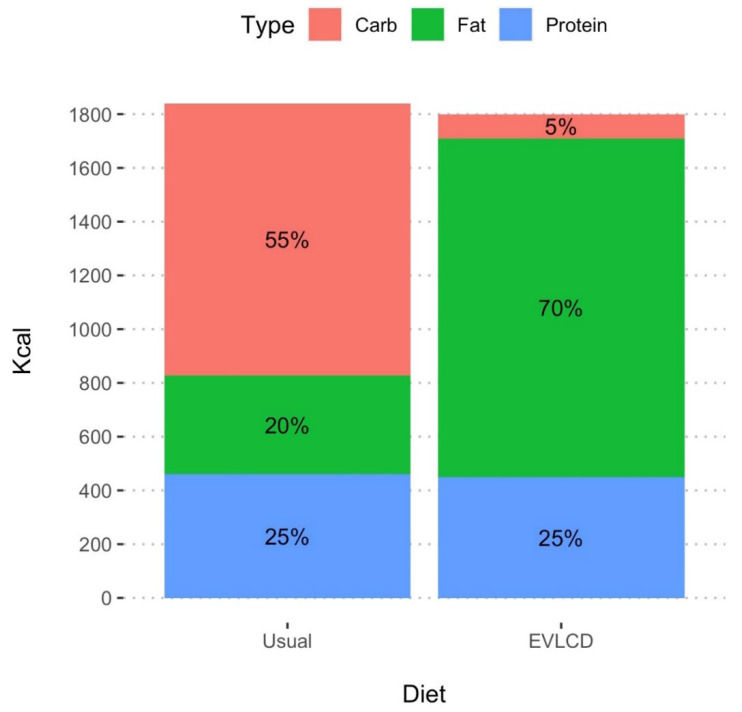
Visual representation of the total amount of Kcal and of the proportion of carb, fat, and protein in usual diet and in EVLCD.

The characteristics of the eating patterns (usual diet and eucaloric very low-carb diet) in terms of percentages of macronutrients are shown in Figure 2.

Total daily energy expenditure (TDEE) was valuated starting from the estimation of the basal metabolic rate (BMR) through the Mifflin formula and multiplying it by the presumed level of physical activity [25].

The EVLCD, prescribed and calculated by a physician expert in nutrition, included mainly vegetables, olive oil, fish, white meat, eggs, nuts, butter and cheese. To maximize dietary variety and adherence to the diet, the diet was integrated with low-carb food products (bread, pasta, rice, rusks, and sweets, with a carbohydrate content lower than 5%). The data about food assumption were collected by the patients themselves daily for the entire year and provided to the investigators monthly.

### 2.4. Insulin Therapy

At baseline, all patients were treated with multiple daily injections of insulin therapy: mean rapid-acting insulin at meals of 18 ± 9.5 IU 18.3 ± 8.0(IRQ: 13.0, 24.0) IU of basal insulin. Basal insulin and rapid insulin were respectively 50% of total daily insulin, and rapid insulin was divided among breakfast, lunch and dinner. The mean units of insulin per kg at baseline were 0.54 ± 0.22 IU/kg/day. No patient at baseline was treated with oral antidiabetic therapy. No patient was treated with continuous subcutaneous insulin infusion.

When patients switched to EVLCD, they were treated with multiple daily injections of insulin therapy, mean rapid- acting insulin of 10.3± 6.5 IU and 18.6± 5.1 IU of basal insulin. Basal insulin was set approximately at 70% of total daily insulin. Rapid insulin was set approximately at 30% of total daily insulin, divided among breakfast, lunch and dinner. The insulin regimen was prescribed at baseline exclusively on body weight 0.42 ± 0.12 (IU/kg/day. No carb counting and no insulin–carb ratio was calculated. Depending on glycemic response, insulin therapy was properly titrated through the use of telephone or messaging applications.

### 2.5. Statistical Analysis

Continuous variables were summarized with mean and standard deviation; categorical variables were summarized with count and percentage. Pairwise Mann–Whitney test was performed to detect the continuous parameters with a significant before–after change, while McNemar’s chi-squared test was used for categorical variables.

BMI and HbA1c levels were categorized into groups and treated as both continuous and pseudo-continuous variables.

HbA1c variance at the two timepoints was compared with the F test, and the reported F value was interpretable as the estimated ratio of the two variances. The pairwise variation between before and after measurements was reported with mean difference and 95% confidence interval (95% CI).

Two-tailed *p* values below 0.05 were considered significant.

Statistical analysis was conducted using IBM SPSS Statistics, version 25.0 (SPSS Inc., Chicago, IL, USA) and R software version 4.2.0.

### 2.6. Ethical Approval Statement

Informed written consent for the use of personal data was obtained from patients.

The study was conducted in accordance with the Declaration of Helsinki and was approved by the Ethics Committee (register number CER Liguria: 365/2022-DB id 12515).

## 3. Results

Enrolled patients were, on average, young subjects (mean age 41.6 ± 15.0 years), predominantly females (69.7%), affected by long-term DM 1 (mean years 14.3 ± 11.3) (Table 1).

At baseline, the mean BMI was 23.9 ± 3.6 kg/m^2^; 72.7% of subjects were normal weight, 21.2% were overweight, and 6.1% were individuals with class I obesity. No cases of higher classes of obesity were present. At the endline, the mean BMI was 24.1 ± 3.1 kg/m^2^; 78.8% of subjects were normal weight, 18.2% were overweight and 3.0% had class I obesity. Two overweight subjects became normal weighted and one obese subject of type I became overweight.

At baseline, the mean systolic arterial pressure was 129.6 ± 7.8 mmHg, and the mean diastolic pressure was 79.1 ± 5.1 mmHg.

At the endline, the mean systolic arterial pressure was 128.6 ± 10.1 mmHg, and the mean diastolic pressure was 79.0 ± 5.3 mmHg.

At baseline, the mean LDL cholesterol level was 98.5 ± 36.1 mg/dL, the mean high-density lipoprotein (HDL) cholesterol was 67.0 ± 14.5 mg/dL, and the main triglyceride level was 74.2 ± 31.8 mg/dL. The glomerular filtration rate (GFR) was 95.7 ± 20.9 mL/min.

At the endline, the mean LDL cholesterol level was 84.4 ± 26.2 mg/dL, the mean high-density lipoprotein (HDL) cholesterol was 71.3 ± 18.9 mg/dL, and the main triglyceride level was 70.4 ± 28.9 mg/dL. The glomerular filtration rate (GFR) was 94.2 ± 20.6 mL/min (Table 2).

No significant improvements in any of the parameters considered above were observed, except for a significant improvement in LDL level (*p* = 0.005).

Antropometrical data, pressure and biochemicals at baseline and at endline are reported in Table 2.

In terms of CV risk factors, at baseline, no patient reported a history of major cardiovascular event, 15% of patients were affected by atherosclerotic carotid disease, 15% were active smokers, and 9% were affected by hypertension. In terms of diabetes microvascular complications, analyzing the medical recordings, at baseline, 18% suffered from diabetic retinopathy, 6% from diabetic nephropathy and 3% from diabetic neuropathy.

At the endline, upon ophthalmologist re-evaluation, no cases of new onset retinopathy were reported. No cardiovascular events and no new cases of nephropathy and neuropathy were reported during follow-ups.

At baseline, 66% of the patients were treated with statin (atorvastatin 40 mg or rosuvastatin 20 mg) and the therapy prescription remained the same for the entire duration of the diet.

At baseline, the mean glycated hemoglobin (HbA1c) was 8.34 ± 1.73%; 12% of the subjects had an HbA1c level <7%, 33% had an HbA1c level between 7 and 8% included, 33% had an HbA1c level between 8 and 9%, and 21% had an HbA1c level >9%.

At the endline, the mean glycated hemoglobin was 6.8 ± 0.78%; 57% of subjects reached a value of HbA1c <7%, 40% a value of HbA1c between 7% and 8%, 3% a value of HbA1c between 8% and 9% included, and no patient had a value of HbA1c >9%. In only one case (3%), the value of glycated hemoglobin worsened from 7.3% to 7.6%.

To highlight, the mean glycated hemoglobin (HbA1c) lowered from 8.3 ± 1.7% to 6.8 ± 0.8 1 subject worsened the HbA1c levels, 1 remained stable and the other 31 improved.

**Figure 3:**
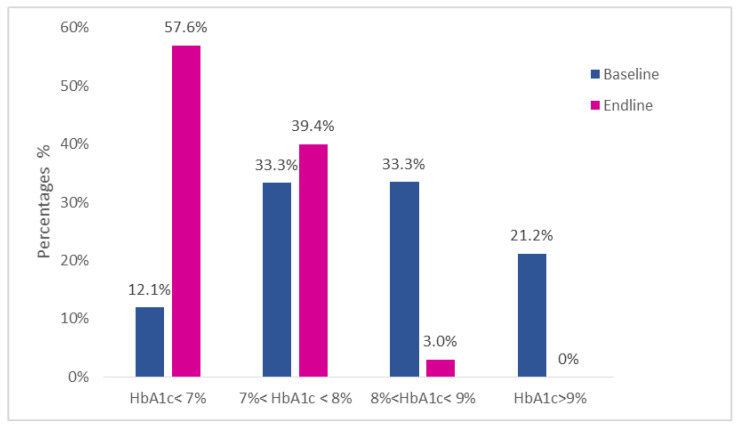
Percentage distribution of the glycated hemoglobin classes of the 33 patients of the study.

Distribution of the different percentage classes of glycated hemoglobin of the patients before and after the diet are shown in Table 3 and Figure 3.

About insulin titration, the dosage of rapid-acting insulin lowered from 18.3 ± 9.5 UI/day to 10.3 ± 6.5 UI/day with a level of significance *p* < 0.001. Consequently, even a reduction statistically significant (*p* < 0.001) in total daily insulin units was observed (from 36.7 ± 14.9 UI/day to 28.9 ± 9.1 UI/day).

The basal total insulin dosage remained unchanged (from 18.3 ± 8.0 UI/day to 18.6 ± 5.1 UI/day) with a statistically significant (*p* < 0.001) increase in the basal units/total daily units ratio (from 51.0 ± 1.3% to 66.0 ± 1.2%).

At baseline, 54.5% of patients reported at least 1 episode of level 2 hypoglycemia while 30.3% reported at least 1 episode of severe hypoglycemia in the previous 12 months. At endline, seven patients (24.2%) reported one single episode of level 2 hypoglycemia during the diet, and this decrease was statistically significant (*p* < 0.001). No cases of severe hypoglycemia and diabetic ketoacidosis were recorded.

The characteristics of the patients in terms of metabolic control and therapeutic approach are summarized in Table 4.

The estimation of diet adherence was performed through a dedicated application for food diary (MyfitnessPal ^TM^ with verified data).

**Figure 4:**
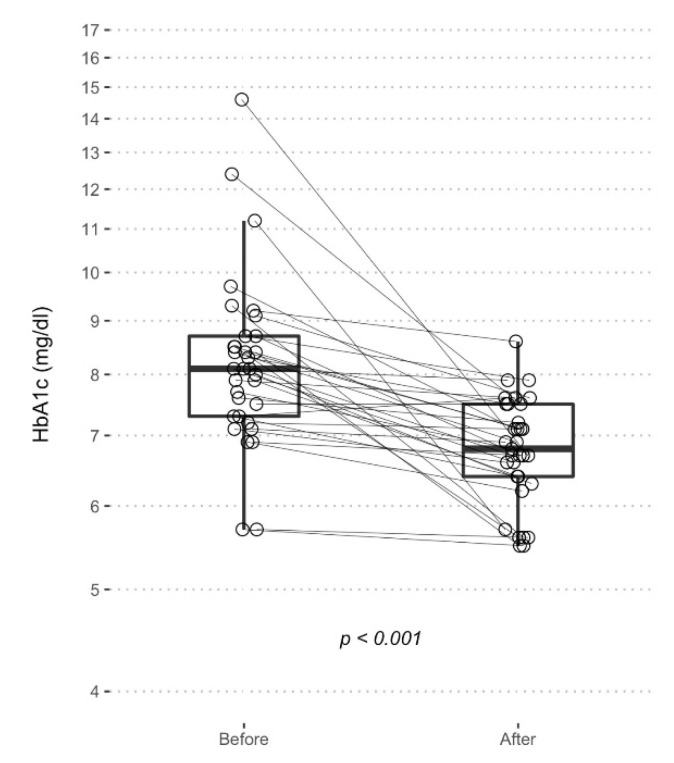
Boxplot represents HbA1c levels before and after EVLCD. Each point represents a single measurement, and the solid lines connects the two measurements of each patient.

Moreover, there was a reduction in the variance of the HbA1c (F = 4.87, *p* < 0.001) from a standard deviation of 1.73% and a coefficient of variation of 21%, indicative of a relevant inhomogeneity in the glycemic control (Figure 2) to a standard deviation of 0.78% and a coefficient of variation of 11%, which indicates less variability in the clinical response of the insulin therapy (Figure 4).

## 4. Discussion

In this retrospective study, 33 patients with type 1 diabetes mellitus, regularly treated with multiple daily injection (MDI) insulin therapy, switched from their usual diet (high-carb, low fat) to an EVLCD (high fat, low carb).

Carbohydrate restriction required a concomitant reduction in the absolute number of insulin units, especially rapid insulin, which is more involved in the management of postprandial hyperglycemia; the low carbohydrate intake also led to a reduction in postprandial glycemic peaks, reducing the glycemic average, glycemic variability, and the insulinogenic stimulus at the level of residual beta cells. This has been shown to be in line with other studies on the subject [21,23].

In terms of clinical outcomes, dietary changes proved to be extremely effective in containing glycemic averages (mean glycemic hemoglobin went from 8.3% to 6.8%) and in reaching the therapeutic target, with an increase in patients with glycated hemoglobin <7% of about +475%. The drastic reduction of patients in very poor control (patients with glycated hemoglobin >8% went from 54.5% to 3%) is remarkable as well. This last result could be very impacting on DM1 considering the results of the DCCT-EDIC study, which demonstrated how the greater improvement in terms of microvascular complications occurs when passing from very poor to fairy control [26]. It should also be noted that the improvement in glycated hemoglobin values was transversal among patients (32 of the 33 patients in the study improved), as shown by a drastic inter-patient reduction in the standard deviation of glycated hemoglobin values. The effectiveness was found to be consistent with other studies on the subject [23,24].

In terms of safety, the reduction of insulin units required for a low-carb diet played an important role in containing hypoglycemic events, with a halving of level II hypoglycemia compared to the previous diet and a total absence of severe hypoglycemic events. Although the microsecretory beta-cellular activity of individual patients is not known, there is scientific evidence that this is present throughout the life of patients with DM1 [27], and that it is stimulated, in particular, by hyperglycemic meals; therefore it’s possible to hypothesize that the reduction of hypoglycemia events derive not only by a lower exogenous insulin units injection but also from a lower production of endogenous insulin as response to a high carb meal.

As shown by the recent literature, the use of many insulin units in DM1 management is linked to a cancer risk 4 times higher over time, with a lower risk when the insulin dosage is below 0.5 UI/kg/die [28]. The reduction in insulin dosage to 0.43 UI/kg/die as observed in our study could be also appropriate in terms of cancer risk management.

No cases of diabetic ketoacidosis were observed; in the literature, in fact, ketoacidosis does not seem physiopathologically attributable to low carbohydrate diets but to severe hypoinsulinemia and hyperglycemia [13], which did not occur here.

From a physiopathological point of view, the effectiveness and safety of EVLCD could be explained as patients assumed too few carbohydrates to develop marked hyperglycemia and too few insulin units to develop marked hypoglycemia, achieving a satisfactory overall control of the disease through the limitations of the variables involved in the pathophysiology of the disease itself.

The significant improvement in LDL observed appears to be due to the greater adherence to statin therapy than to diet itself. There were no significant changes in other parameters or biomarkers.

The study has several limitations.

First, the patients voluntarily decided to switch from their usual diet (high-carb, low fat) to an EVLCD (low carb, high fat), motivated by the glycemic stability that this nutritional plan generally entails. It is therefore a group of highly motivated patients, willing to change their diet and maintain an EVLCD over time. Results may not be as good in less motivated patients.

Secondly, although in the literature studies like this are very limited, and when present they are often case series, the number of patients is very small. Moreover, it is also a retrospective study, not a randomized one with a control group.

Finally, unlike other experiences published in the literature, a very close telematic follow-up was performed here. Although in our clinical practice we perform the same monitoring in all our patients, regardless of the carbohydrate content of the diet, it is possible that, without such medical supervision, the results may be less satisfactory.

Despite the obvious limitations, the results in terms of efficacy and safety seem very promising and we hope that these provide a starting point for further research on the nutritional plan adopted.

## 5. Conclusions

Although nowadays (VLCD) are not routinely proposed to DM 1 patients as an alternative nutritional plan, this retrospective study has shown that they seem safe and effective if adopted under tight medical control with a proper adjustment in insulin therapy.

Long-term outcomes of VLCD in patients affected by DM 1 are unknown and still debated. In light of the literature published today, our opinion is that randomized control trials on the topic are mandatory.

## Figures and Tables

**Table 1:** Demographic characteristics of the patients of the study. * N (%) or mean ± standard deviation.

Feature	Value *
Number of Patients	33
Number of Females	23 (69.7%)
Age (y)	41.6 ± 15.0
Years of Diabetes	14.3 ± 11.3

**Table 2:** Clinical and biochemical data of the patients before and after the EVLCD.

	Before *n* = 33	After *n* = 33	Variation	
			Mean (95% CI)	p *
Weight (kg)	68.9 (13.5)	66 (60, 80)	0.36 (−0.62, 1.35)	*0.456*
BMI (kg/m^2^)	23.9 (3.6)	24.1 (3.1)	0.14 (−0.20, 0.48)	*0.419*
BMI group, N (%)				*0.083 §*
<25 kg/m^2^	24 (72.7)	26 (78.8)	-	
25–30 kg/m^2^	7 (21.2)	6 (18.2)		
30–35 kg/m^2^	2 (6.1)	1 (3.0)		
SBP (mmHg)	129.6 (7.8)	128.6 (10.1)	−0.97 (−3.80, 1.86)	*0.491*
DBP (mmHg)	79.1 (5.1)	79.0 (5.3)	−0.09 (−2.22, 2.04)	*0.931*
Total cholesterol (mg/dL)	180.3 (36.2)	169.8 (38.4)	−10.6 (−21.3, 0.1)	*0.052*
LDL (mg/dL)	98.5 (36.1)	84.4 (26.2)	−14.1 (−23.6, −4.6)	*0.005*
HDL (mg/dL)	67.0 (14.5)	71.3 (18.9)	4.27 (0.00, 8.55)	*0.050*
Non-HDL (mg/dL)	113.3 (36.4)	98.5 (27.3)	−14.9 (−24.4, −5.3)	*0.003*
LDL/HDR ratio	1.6 (0.8)	1.2 (0.4)	−0.33 (−0.52, −0.13)	*0.002*
TG (mg/dL)	74.2 (31.8)	70.4 (28.9)	−3.73 (−8.02, 0.57)	*0.087*
TG/HDR ratio	1.2 (0.6)	1.1 (0.5)	−0.11 (−0.25, 0.02)	*0.097*
CKD–EPI (ml/min)	95.7 (20.9)	94.2 (20.6)	−1.58 (−3.32, 0.17)	*0.075*

BMI: body mass index; SBP: systolic blood pressure, DBP: diastolic blood pressure; LDL: low-density lipoprotein; HDL: high-density lipoprotein; TG: triglycerides; CDK-EPI: glomerular filtration rate; * paired *t*-test *p* value; § McNemar’s chi-squared test *p* value. There were no missing data: all variables were collected for 33 subjects at two timepoints. Continuous variables are summarized with mean and standard deviation. Categorical variables are summarized with count (N) or percentage (%). Variations with *p* values below 0.05 were considered statistically significant.

**Table 3:** Number and percentage of subjects by HbA1c before and after the EVLCD.

*N* (%)		HbA1c After			
		<7 (mg/dL)	7–8 (mg/dL)	8–9 (mg/dL)	>9 (mg/dL)
**HbA1c Before**	**<7 (mg/dL)**	4 (12.1)	0 (0.0)	0 (0.0)	0 (0.0)
	**7–8 (mg/dL)**	6 (18.2)	5 (15.2)	0 (0.0)	0 (0.0)
	**8–9 (mg/dL)**	6 (18.2)	5 (15.2)	0 (0.0)	0 (0.0)
	**>9 (mg/dL)**	3 (9.1)	3 (9.1)	1 (3.0)	0 (0.0)

Table cells with subjects having the same, lower or higher HbA1c levels after EVLCD compared to before EVLCD are filled in dark gray, light gray and white, respectively. Total number of subjects = 33.

**Table 4:** Glycometabolic control and therapeutic data of the patients before and after EVLCD. Continuous variables are summarized with mean and standard deviation. Categorical variation are summarized as count (N) or percentage (%).

	Before, *n* = 33	After *n* = 33	Variation	
			Mean (95% CI)	*p* *
% HbA1c	8.3 (1.7)	6.8 (0.8)	−1.54 (−2.17, −0.91)	<0.001
HbA1c group, N (%)			-	0.001
<7%	4 (12.1)	19 (57.6)		
7 to 8%	11 (33.3)	13 (39.4)		
8 to 9%	11 (33.3)	1 (3.0)		
>9%	7 (21.2)	0 (0.0)		
Level 2 Hypoglycemia, N (%)	18 (54.5)	8 (24.2)	-	0.034
Severe Hypoglycemia, N (%)	10 (30.3)	0 (0)	-	<0.001
IU Total Insulin	36.7 (14.9)	28.9 (9.1)	−7.7 (−11.0, −4.5)	<0.001
IU/kg/day	0.54 (0.22)	0.42 (0.12)	−0.12 (−0.17, −0.07)	<0.001
IU Rapid-acting Insulin	18.3 (9.5)	10.3 (6.5)	-8.0 (−10.3, −5.7)	<0.001
% Rapid-acting Insulin	49 (13)	34 (12)	−15 (−19, −11)	<0.001
IU Basal Insulin	18.3 (8.0)	18.6 (5.1)	0.3 (−1.5, 2.1)	0.760
% Basal Insulin	0.51 (0.13)	0.66 (0.12)	15 (11, 19)	<0.001

HbA1c: glycated hemoglobin; UI: unit of insulin; * paired *t*-test *p* value; McNemar’s chi-squared test *p* value. There were no missing data: all variables were collected for 33 subjects at two timepoints. Variations with *p* values below 0.05 were considered statistically significant.

## Data Availability

Not applicable.

## References

[1] Atkinson M.A., Maclaren N.K. (1994). The pathogenesis of insulin-dependent diabetes mellitus. N. Engl. J. Med..

[2] Zaccardi F., Webb D.R., Yates T., Davies M.J. (2016). Pathophysiology of type 1 and type 2 diabetes mellitus: A 90-year perspective. Postgrad. Med. J..

[3] Westman E.C., Yancy W.S., Humphreys M. (2006). Dietary treatment of diabetes mellitus in the pre-insulin era (1914-1922). Perspect. Biol. Med..

[4] Nathan D.M., Cleary P.A., Backlund J.Y., Genuth S.M., Lachin J.M., Orchard T.J., Raskin P., Zinman B. (2005). Diabetes Control and Complications Trial/Epidemiology of Diabetes Interventions and Complications (DCCT/EDIC) Study Research Group. Intensive diabetes treatment and cardiovascular disease in patients with type 1 diabetes. N. Engl. J. Med..

[5] Ewers B., Vilsbøll T., Andersen H.U., Bruun J.M. (2019). The dietary education trial in carbohydrate counting (DIET-CARB Study): Study protocol for a randomised, parallel, open-label, intervention study comparing different approaches to dietary self-management in patients with type 1 diabetes. BMJ Open.

[6] Barnard-Kelly K.D., Naranjo D., Majidi S., Akturk H.K., Breton M., Courtet P., Olié E., Lal R.A., Johnson N., Renard E. (2020). Suicide and Self-inflicted Injury in Diabetes: A Balancing Act. J. Diabetes Sci Technol..

[7] Mair C., Wulaningsih W., Jeyam A., McGurnaghan S., Blackbourn L., Kennon B., Leese G., Lindsay R., McCrimmon R.J., McKnight J. (2019). Scottish Diabetes Research Network (SDRN) Epidemiology Group. Glycaemic control trends in people with type 1 diabetes in Scotland 2004-2016. Diabetologia.

[8] Gruppo di studio ANNALI AMD (2011). Gli Annali AMD: Un modello di monitoraggio sistematico e miglioramento continuo della qualità dell’assistenza diabetologica [AMD Annals: A model of continuous monitoring and improvement of the quality of diabetes care]. Epidemiol. Prev..

[9] Lam-Chung C.E., Martínez Zavala N., Ibarra-Salce R., Pozos Varela F.J., Mena Ureta T.S., Berumen Hermosillo F., Campos Muñoz A., Janka Zires M., Almeda-Valdes P. (2021). Association of estimated glucose disposal rate and chronic diabetic complications in patients with type 1 diabetes. Endocrinol. Diabetes Metab..

[10] Rawshani A., Rawshani A., Franzén S., Eliasson B., Svensson A.M., Miftaraj M., McGuire D.K., Sattar N., Rosengren A., Gudbjörnsdottir S. (2017). Mortality and Cardiovascular Disease in Type 1 and Type 2 Diabetes. N. Engl. J. Med..

[11] Iqbal A., Heller S.R. (2018). The role of structured education in the management of hypoglycaemia. Diabetologia.

[12] Driscoll K.A., Raymond J., Naranjo D., Patton S.R. (2016). Fear of Hypoglycemia in Children and Adolescents and Their Parents with Type 1 Diabetes. Curr. Diab. Rep..

[13] Fazeli Farsani S., Brodovicz K., Soleymanlou N., Marquard J., Wissinger E., Maiese B.A. (2017). Incidence and prevalence of diabetic ketoacidosis (DKA) among adults with type 1 diabetes mellitus (T1D): A systematic literature review. BMJ Open.

[14] Zhong V.W., Juhaeri J., Cole S.R., Kontopantelis E., Shay C.M., Gordon-Larsen P., Mayer-Davis E.J. (2017). Incidence and Trends in Hypoglycemia Hospitalization in Adults with Type 1 and Type 2 Diabetes in England, 1998-2013: A Retrospective Cohort Study. Diabetes Care.

[15] Zhong V.W., Juhaeri J., Mayer-Davis E.J. (2018). Trends in Hospital Admission for Diabetic Ketoacidosis in Adults with Type 1 and Type 2 Diabetes in England, 1998-2013: A Retrospective Cohort Study. Diabetes Care.

[16] Bronstone A., Graham C. (2016). The Potential Cost Implications of Averting Severe Hypoglycemic Events Requiring Hospitalization in High-Risk Adults with Type 1 Diabetes Using Real-Time Continuous Glucose Monitoring. J. Diabetes Sci. Technol..

[17] Jensen M.H., Dethlefsen C., Hejlesen O., Vestergaard P. (2020). Association of severe hypoglycemia with mortality for people with diabetes mellitus during a 20-year follow-up in Denmark: A cohort study. Acta Diabetol..

[18] Benoit S.R., Zhang Y., Geiss L.S., Gregg E.W., Albright A. (2018). Trends in Diabetic Ketoacidosis Hospitalizations and In-Hospital Mortality-United States, 2000-2014. MMWR Morb. Mortal Wkly. Rep..

[19] Sukkar S.G., Muscaritoli M. (2021). A Clinical Perspective of Low Carbohydrate Ketogenic Diets: A Narrative Review. Front. Nutr..

[20] Mike H. An Interview with Low-Carb Pioneer Dr. Richard Bernstein. https://www.healthline.com/diabetesmine/interview-low-carb-pioneer-dr-richard-bernstein#1.

[21] Turton J.L., Raab R., Rooney K.B. (2018). Low-carbohydrate diets for type 1 diabetes mellitus: A systematic review. PLoS ONE.

[22] Nielsen J.V., Gando C., Joensson E., Paulsson C. (2012). Low carbohydrate diet in type 1 diabetes, long-term improvement and adherence: A clinical audit. Diabetol. Metab. Syndr..

[23] Lennerz B.S., Barton A., Bernstein R.K., Dikeman R.D., Diulus C., Hallberg S., Rhodes E.T., Ebbeling C.B., Westman E.C., Yancy W.S. (2018). Management of Type 1 Diabetes with a Very Low-Carbohydrate Diet. Pediatrics.

[24] Bolla A.M., Caretto A., Laurenzi A., Scavini M., Piemonti L. (2019). Low-Carb and Ketogenic Diets in Type 1 and Type 2 Diabetes. Nutrients.

[25] Scalfi L., Censi L., Marra M., Maffeis C., Pecoraro P., Polito A., Strata A., Tagliabue A. (2014). Nutrients and Energy Reference Intake for Italian Population.

[26] Diabetes Control and Complications Trial (DCCT), Epidemiology of Diabetes Interventions and Complications (EDIC) Study Research Group (2016). Intensive Diabetes Treatment and Cardiovascular Outcomes in Type 1 Diabetes: The DCCT/EDIC Study 30-Year Follow-up. Diabetes Care.

[27] Oram R.A., Jones A.G., Besser R.E.J., Knight B.A., Shields B.M., Brown R.J., Hattersley A.T., McDonald T.J. (2014). The majority of patients with long-duration type 1 diabetes are insulin microsecretors and have functioning beta cells. Diabetologia.

[28] Zhong W., Mao Y. (2022). Daily Insulin Dose and Cancer Risk Among Patients with Type 1 Diabetes. JAMA Oncol..

